# The responsiveness of criminal networks to intentional attacks: Disrupting darknet drug trade

**DOI:** 10.1371/journal.pone.0238019

**Published:** 2020-09-10

**Authors:** Scott Duxbury, Dana L. Haynie

**Affiliations:** 1 Department of Sociology, University of North Carolina, Chapel Hill, North Carolina, United States of America; 2 Department of Sociology, The Ohio State University, Columbus, Ohio, United States of America; Central European University, HUNGARY

## Abstract

Physical, technological, and social networks are often at risk of intentional attack. Despite the wide-spanning importance of network vulnerability, very little is known about how criminal networks respond to attacks or whether intentional attacks affect criminal activity in the long-run. To assess criminal network responsiveness, we designed an empirically-grounded agent-based simulation using population-level network data on 16,847 illicit drug exchanges between 7,295 users of an active darknet drug market and statistical methods for simulation analysis. We consider three attack strategies: targeted attacks that delete structurally integral vertices, weak link attacks that delete large numbers of weakly connected vertices, and signal attacks that saturate the network with noisy signals. Results reveal that, while targeted attacks are effective when conducted at a large-scale, weak link and signal attacks deter more potential drug transactions and buyers when only a small portion of the network is attacked. We also find that intentional attacks affect network behavior. When networks are attacked, actors grow more cautious about forging ties, connecting less frequently and only to trustworthy alters. Operating in tandem, these two processes undermine long-term network robustness and increase network vulnerability to future attacks.

## Introduction

As human societies grow increasingly complex and interdependent, they become more reliant on technological, infrastructural, and social networks. This greater reliance on networks has resulted in growing concern about network vulnerability: a network’s ability to weather intentional attacks [1–15]. Network vulnerability has been the subject of much interdisciplinary inquiry and findings from this body of research have contributed to diverse areas of science. To physics, by providing analytic insight to the statistical mechanics of complex networks [1, 7, 11, 15]. To medicine, by evaluating points of intervention to impede disease diffusion [16]. To biology, by assessing the resilience of animal herds to the loss of members [17]. And, to social science, by offering insight to group dynamics in unstable environments [18, 19].

Despite the far-reaching importance of this topic, social scientists know little about social network responsiveness: how social networks react to and recover from intentional attacks. Most research has examined static networks compiled from cross-sectional data [1, 2, 7, 9, 11, 14].

Yet, given that social networks contain human actors who are capable of rational behavior, it is likely that social networks adapt to attacks and possible that actors strive to insulate the network from additional damage. Moreover, prior studies have largely assessed attack strategies that isolate and remove structurally integral vertices—vertices that are highly connected or broker otherwise disjointed network components. Since attackers rarely have complete information on network structure or easy-access to influential actors [7, 15, 19], these findings tell us little about the vulnerability or responsiveness of social networks to commonly used diffuse attack strategies which target the network at large, rather than a few focal actors.

These limitations are especially pressing in research on criminological networks (e.g., gangs, drug markets), where structurally integral actors are usually inaccessible, most network behavior is hidden, actors have strong incentives to limit periods of inactivity, and findings bear on public well-being [18–21]. Data on criminal networks also tend to come from court or police records of captured crime rings [9, 10, 14, 19–22], meaning that findings from current research are biased towards inactive and “unsuccessful” networks. Moreover, while crimes like homicide and motor vehicle theft are generally well recorded, most other index offense crimes including robbery, larceny, rape, assault, and drug crimes are under reported, and thus, data on crime occurrence—either from self-report or official records—are often inaccurate. Consequently, it is difficult to trace future levels of criminal activity to successful network attacks in the past.

Further, data on criminal networks are limited in availability, and, as a result, past studies have been forced to rely on metrics of criminal interaction that indirectly measure the mechanics of criminal behavior [23, 24]. For instance, Natarajan [25] studied the structure of a heroin distribution network using wiretapped telecommunications. McGloin [22] aggregated over multiple types of affiliation data recorded during semi-structured interviews with police officers to construct a network of gang affiliation, including co-offending and having spent time together in prison. Criminal network data is also usually incomplete, necessitating researchers to assume that the observed network segment is an adequate representation of the unmeasured criminal network. Yet, to pinpoint how criminal networks respond to attacks and to assess the consequences of intentional attacks for crime occurrence, it is essential to observe an entire criminal network over a substantial length of time with accurate information on each participant’s involvement in crime.

To overcome these limitations, we make use of population-level data on a large darknet drug market observed over 14 months. Darknet drug markets are online marketplaces that can be accessed using anonymizing web services to purchase illicit drugs (S1 Appendix, Data). These data have numerous advantages. First, unlike data obtained from surveys or official records, these data are collected through observations of a currently-active criminal network; thus, they contain accurate reports of each market actor’s involvement in illicit drug trade. Second, since all drug transactions are recorded, we observe the entire population of drug exchanges from the birth of the market to the end of data collection. Third, data are collected digitally, so the growth of the market can be observed as it unfolds in real-time. Fourth, online drug exchange is growing more prevalent, as it connects drug distributors and consumers across the globe [26–29]. Thus, it is not only methodologically fruitful to examine online drug trade, but informative of an increasingly common form of crime.

Data for our study come from one of the largest currently operating darknet drug markets, *Silk Road 3*.*1*. They contain information on 16,847 illicit drug transactions between 7,126 buyers and 169 vendors, representing the entire population of drug transactions on the *Silk Road 3*.*1* during its first 14 months of activity (S1 Table in S1 Appendix). From these data, we reconstructed a bipartite network, where a tie connects a buyer and a vendor if the buyer has purchased drugs from that vendor. Next, we designed an empirically grounded agent-based model [30–32]. We discuss the assumptions of the agent-based model in some detail in the S1 Appendix. We first used stochastic actor-oriented models to evaluate why buyers purchase from specific vendors in the *Silk Road 3*.*1* drug exchange network (Model 1, S2 Table in S1 Appendix). We then used the coefficients obtained from stochastic actor-oriented modeling to inform agent decision- making in the simulation. The result is an agent-based simulation with empirically validated rules for agent behavior (*Materials and Methods*). The simulated outcome networks represent the agent-based model’s best estimates of the *Silk Road 3*.*1* drug market at its last moment of observation. By manipulating characteristics of the raw data and repeating the simulation, we can evaluate the effect of various attack strategies on network development.

We consider three attack strategies: targeted attacks, weak link attacks, and signal attacks. Targeted attacks are those that remove structurally integral vertices. Consistent with prior research [2, 9–11, 14], we operationalized targeted attacks by deleting highly connected vendors from the market (Table 1). Our second attack strategy is a weak link attack, where we delete large numbers of weakly connected actors at once. This attack strategy is one that is often used to police open-air drug markets, where numerous low-level drug dealers and users are arrested in quick succession [33]. Our third attack strategy is the signal attack. Signal attacks impede network development by saturating a network with noisy signals. The rise of social media has made signal attacks more common. As an example, social media giants like *Facebook* and *Twitter* have recently struggled to insulate their online platforms against the spread of political propaganda [34], and online markets like *eBay* routinely grapple with opinion spamming through fake sales reviews [35]. We implement signal attacks by reducing the number of positive sales ratings a vendor has received. We conducted each attack strategy at four levels of intervention, reflecting increasingly aggressive attacks (Table 1). Our control group contains networks which were simulated without any treatment (S1 Fig in S1 Appendix). With one control condition and three attack strategies at four levels of intervention, our study includes 12 (3 x 4) treatment conditions and one control group. To ensure that our results are not idiosyncratic to a specific simulated change process, we repeated our agent-based simulation 100 times for each level of the experiment. Across levels of treatment, this yielded a total of 1,300 networks containing 10,213,770 buyers, 255,504 vendors, and 7,060,303 drug exchanges for analysis.

**Table 1:** Experiment design. Degree centrality is the raw sum of ties incident to an actor.

Attack Strategy	Measurement	Level of Intervention			
		Low	Medium-low	Medium-high	High
Targeted attack	Delete vendors in top *n*th percentile of degree centrality.	20th	40th	60th	80th
Weak link attack	Delete buyers in bottom *n*th percentile of degree centrality.	20th	40th	60th	80th
Signal attack	Reduce vendors’ cumulative sales ratings by *n* %	20%	40%	60%	80%

## Results

**Fig 1:**
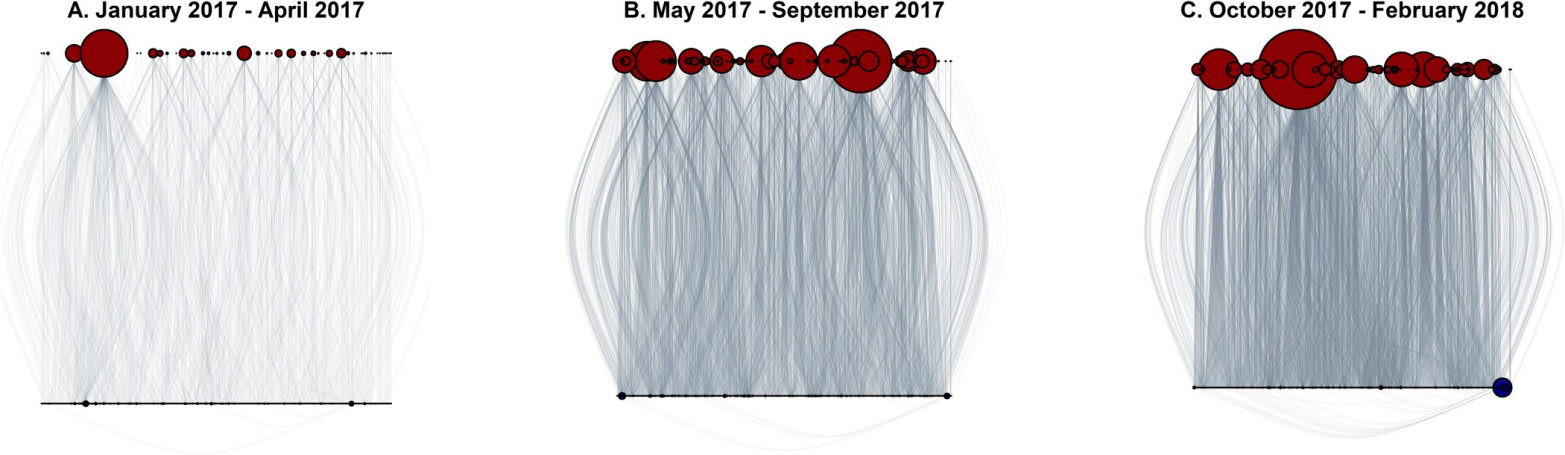
*Silk Road 3*.*1* drug exchange network over time. Blue nodes are buyers, red nodes are vendors, and lines are illicit drug transactions. Node size is proportional to buyer/vendor degree centrality. In Panel A, 𝑛_𝑏𝑢𝑦𝑒𝑟𝑠_ = 505, 𝑛_𝑣𝑒𝑛𝑑𝑜𝑟𝑠_ = 50, 𝑛_𝑡𝑟𝑎𝑛𝑠𝑎𝑐𝑡𝑖𝑜𝑛𝑠_ = 1,110. In Panel B, 𝑛𝑏𝑢𝑦𝑒𝑟𝑠 = 2,977, 𝑑𝑜𝑟𝑠 = 121, 𝑛𝑡𝑟𝑎𝑛𝑠𝑎𝑐𝑡𝑖𝑜𝑛𝑠 = 6,736. In Panel C, 𝑛𝑏𝑢 = 4,323, 𝑛_𝑣𝑒𝑛𝑑𝑜𝑟𝑠_ = 101, 𝑛_𝑡𝑟𝑎𝑛𝑠𝑎𝑐𝑡𝑖𝑜𝑛𝑠_ = 9,001.

Are criminal networks vulnerable to intentional attacks? We first use methods developed in cross-sectional research to provide a baseline for comparing results from agent-based simulation. A common way to assess network vulnerability is to examine preferential attachment—the tendency for buyers to purchase from drug distributors with large degree centrality—since networks with high levels of preferential attachment tend to be vulnerable to targeted attacks [1, 7, 8]. Fig 1 illustrates that there are a handful of highly connected vendors, where 56% of drug transactions involve only 10% of vendors in the aggregate network. We formally assess preferential attachment by calculating the degree-scaling coefficient (S1 Appendix, Measurement), a commonly used indicator of preferential attachment [1, 36, 37]. The degree-scaling coefficient is 1.52 (Kolmogorov-Smirnov statistic = .12, *P* =. 99, null hypothesis is that the degree-scaling coefficient is not zero, S1 Table in S1 Appendix), reflecting high levels of preferential attachment and, thus, vulnerability to intentional attacks. A second strategy for gauging network vulnerability is to compute the degree-degree correlation (S1 Appendix, Measurement), where positive values indicate that the network is robust to intentional attacks and negative values indicate vulnerability [11, 13, 14]. The degree-degree correlation is -.07 (S1 Table in S1 Appendix), suggesting vulnerability. This is supported by vertex deletion simulations showing that the network can be completely dismantled by deleting 35% of the vendors on the market, and 20.3% of buyers are rendered isolates by deleting only 10 vendors (S2 Fig in S1 Appendix). Collectively, results from cross-sectional analyses suggest that the network is vulnerable to intentional attacks and replicate several prior findings on criminal network vulnerability [6, 9, 14, 20]. But, are these conclusions supported when we account for network responsiveness?

The problem of network responsiveness can be characterized by two questions: Do intentional attacks affect network activity in the long run? And, do networks adapt to attacks over time? To address these questions, we turn to results from agent-based simulation.

**Fig 2:**
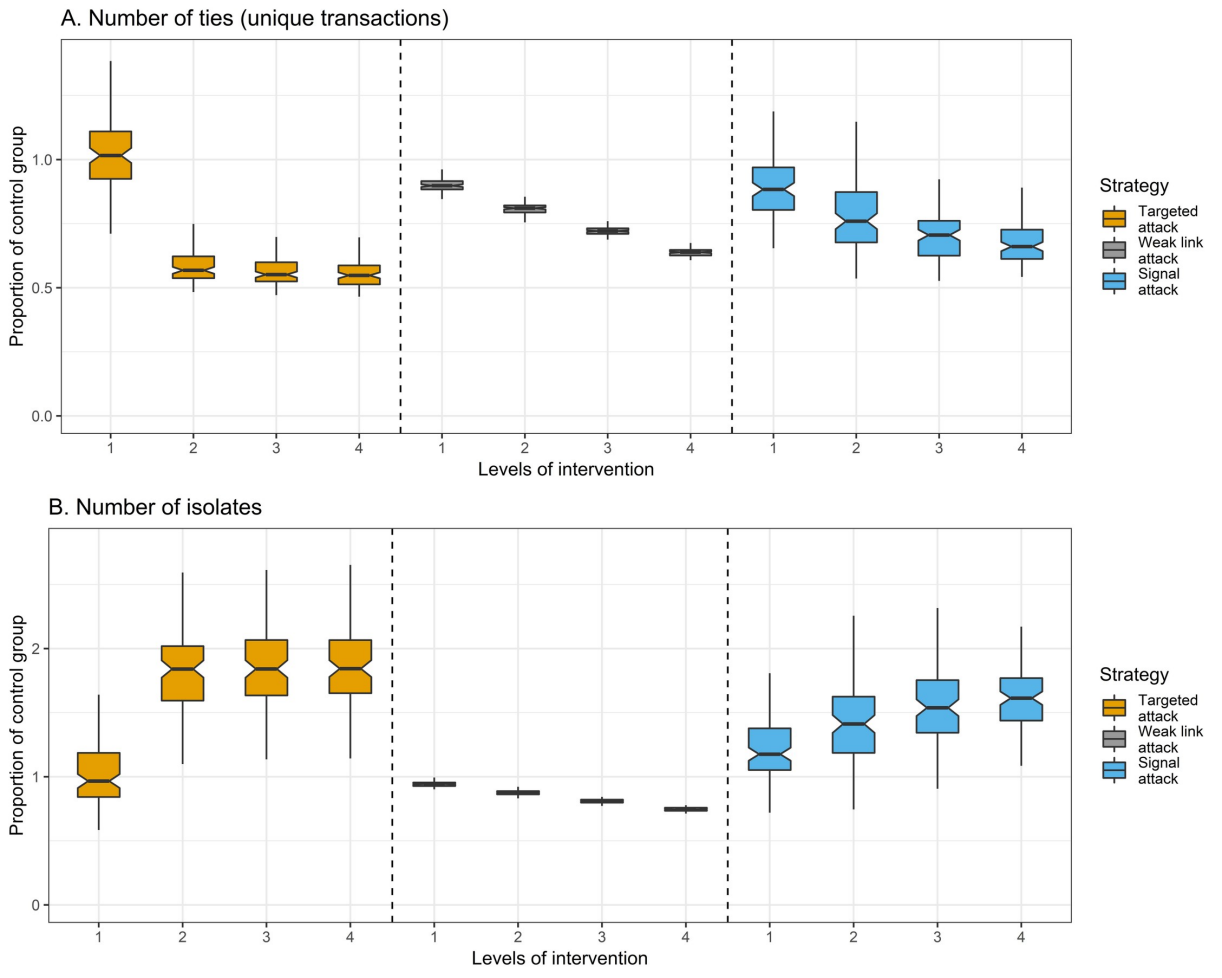
Criminal activity by level of intervention and attack strategy (*n* = 1,200 networks). *Y* axis is proportion of control group (treatment divided by control group). *X* axis is the level of intervention, where a 1 is low intervention, 2 is medium-low, 3 is medium-high, and 4 is high. Boxplots are plotted using the Tukey method. Grey boxplots are targeted attacks (*n* = 400), yellow boxplots are weak link attacks (*n* = 400), blue boxplots are signal attacks (*n* = 400). The correlation between the level of intervention and the number of ties is -.84 (*P* < .001) for the targeted attack strategy, .99 (*P* < .001) for the weak link strategy, and -.79 (*P* < .001) for the signal attack. The correlation between the level of intervention and the number of isolates is -.84 (*P* < .001) for the targeted attack strategy, -.99 (*P* < .001) for the weak link attack strategy, and .79 (*P* < .001) for the signal strategy.

Our first concern is to assess how much criminal activity is deterred by an attack. Fig 2A considers change in the number of illicit drug transactions. There is no significant difference between low-levels of targeted attacks and the control conditions (*t* = -1.01, *P* = .31). However, at the medium-low level of intervention, there is a precipitous decline in the number of drug transactions. After this decrease, the marginal declines in the number of drug transactions are minimal. Weak link attacks and signal attacks tend to reduce the level of drug trafficking more- so than targeted attacks at low levels of intervention. Here, weak link attacks yield a 13.2% decrease in the number of illicit drug exchanges on average, and signal attacks yield a 13.1% decrease, deterring roughly 750 drug exchanges that would have otherwise occurred. This difference reverses at higher levels of intervention, where targeted attacks decrease the number of illicit drug exchanges more-so than either weak link or signal attacks, indicating that targeted attacks are most effective when they are deployed at a large scale. We used ordinary least squares linear regression to assess the mean effect of each attack strategy on the number of drug transactions in a simulated network. Holding the level of intervention constant, weak link attacks prevent, on average, 2,404 drug transactions that would have otherwise occurred (*β* = -2,404, CI = [–2,578, –2,230], *P* < .001, Model 2, S3 Table in S1 Appendix), and targeted attacks prevent 611 drug transactions (*β* = -611, CI = [–640, –571], *P* < .001, Model 2, S3 Table in S1 Appendix). Signal attacks prevent 151 drug transactions that occurred in the control group, but the difference is not significant when controlling for the level of intervention (*β* = -151, CI = [–325, 23], *P* = .09, Model 2, S3 Table in S1 Appendix).

Fig 2B considers a second indicator of criminal activity: the number of isolates.

**Fig 3:**
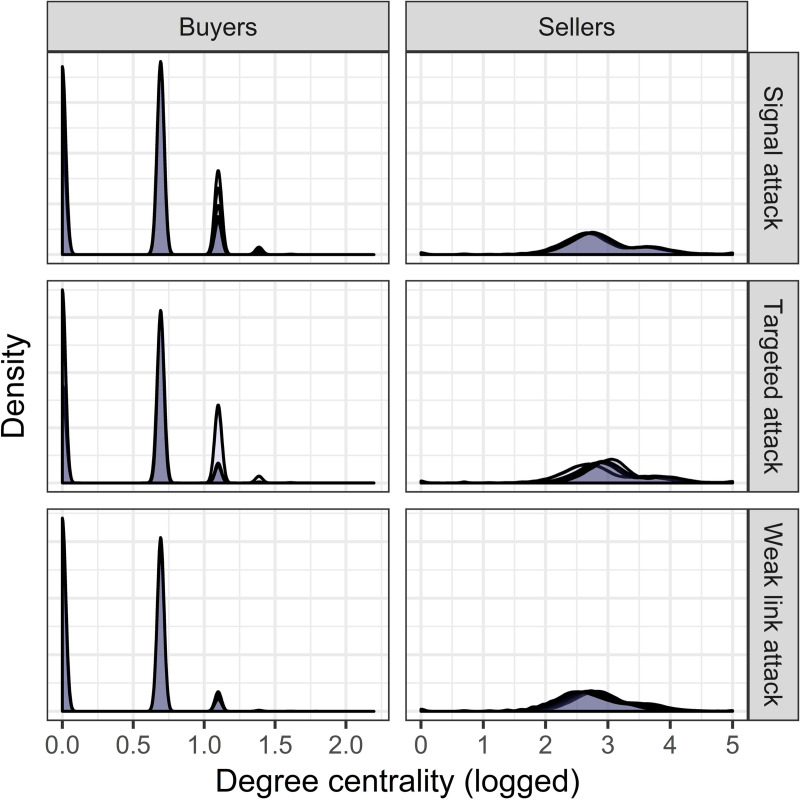
Density plots of buyers’ (*n* = 10,213,770) and vendors’ (*n* = 255,204) degree centrality (logged) by attack strategy. *X* axis is the natural log of degree centrality, *Y* axis is the frequency. Darker shades correspond to higher levels of intervention; lighter shades correspond to lower levels of intervention.

Isolates are actors (typically buyers) who are not connected to any other actor. Increases in the number of isolates reflect actors who would have otherwise purchased illicit drugs but were deterred from doing so. At low levels of intervention, targeted attacks do not substantially affect the number of isolates. In contrast, signal attacks yield a 20.4% increase in the number of isolates—an average of 372 buyers who would have purchased illicit drugs if the network had not been attacked. At higher levels of intervention, signal attacks continue to increase the number of isolates, albeit less-so than targeted attacks. Weak link attacks decrease the number of isolates linearly across levels of intervention. This is because weak link attacks delete those actors who are most likely to be rendered isolates over time, reflecting incapacitation rather than deterrence (S5 Table in S1 Appendix). Across attack strategies, most of the change in criminal activity can be attributed to the behaviors of weakly connected actors. While vendors’ degree centralities are relatively unchanged at higher levels of intervention, increasingly aggressive attacks generate a growing number of isolated and weakly connected buyers (Fig 3). We modeled the probability of becoming an isolate using conditional logistic regression, treating each unique actor as strata and clustering standard errors on networks. Controlling for attack strategy and level of intervention, weakly connected buyers have 70% (𝑒^.529^) higher odds of becoming an isolate than other buyers (*β* = .529, CI = [.529, .529], *P* < .001, Model 3, S4 Table in S1 Appendix), and 57.4 times (𝑒^3.941^) higher odds of becoming an isolate than vendors (*β* = 3.941, CI = [3.941, 3.941], *P* < .001 Model 3, S5 Table in S1 Appendix). This indicates that structurally integral buyers and vendors are relatively unaffected by attacks, while weakly connected buyers are the most likely to be deterred.

Collectively, these results illustrate that weak link and signal attacks reduce more criminal activity at low levels of intervention than targeted attacks, and that weak link attacks are the most effective for reducing criminal activity net of the level of intervention. These findings contrast with cross-sectional research on network vulnerability, which generally finds that most static and non-responsive networks can be dismantled with a relatively small number of targeted nodal deletions [1, 4–6, 8, 15], especially those characterized by high preferential attachment and negative degree-degree correlation [1, 7, 11–14]. Also in contrast to prior research, results indicate that much of the change in criminal activity can be attributed to weakly connected actors’ unwillingness to purchase drugs, rather than the immediate disruptive effect of deleting structurally integral actors.

**Fig 4:**
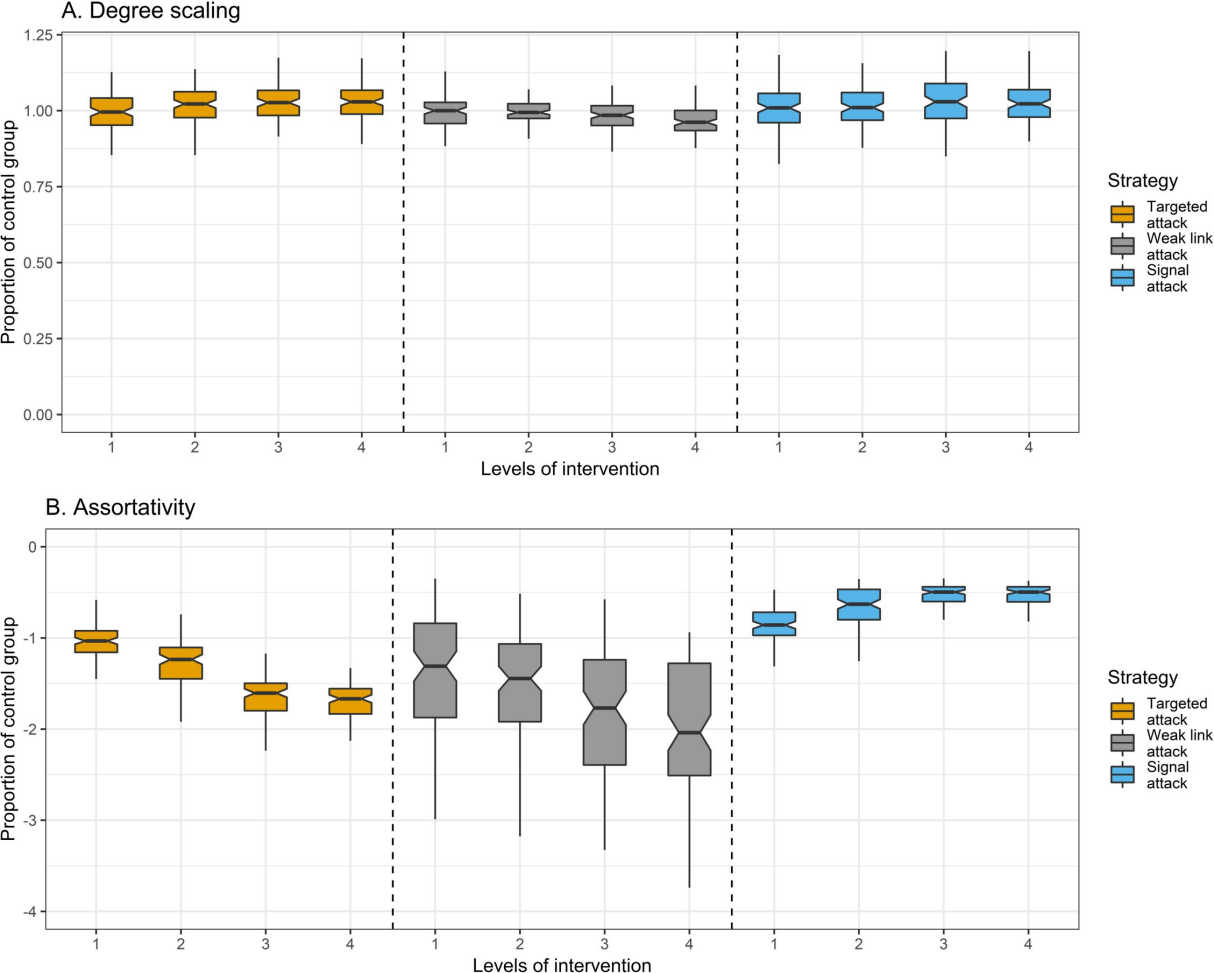
Network behavior by level of intervention and attack strategy (*n* = 1,200 networks). *Y* axis is proportion of control group (treatment divided by control group). *X* axis is the level of intervention, where a 1 is low intervention, 2 is medium-low, 3 is medium-high, and 4 is high. Boxplots are plotted using the Tukey method. Grey boxplots are targeted attacks (*n* = 400), yellow boxplots are weak link attacks (*n* = 400), blue boxplots are signal attacks (*n* = 400). Assortativity is reverse coded to facilitate interpretation since the control-group has negative assortativity (mean = -.32). Thus, -1 is equal to no difference from the control group in Panel B. The correlation between the level of intervention and degree scaling is .26 (*P* < .001) for the targeted attack strategy, -.30 (*P* < .001) for the weak link strategy, and .23 (*P* < .001) for the signal attack. The correlation between the level of intervention and assortativity is -.88 (*P* < .001) for the targeted attack strategy, -.68 (*P* < .001) for the weak link attack strategy, and .76 (*P* < .001) for the signal attack strategy.

**Fig 5:**
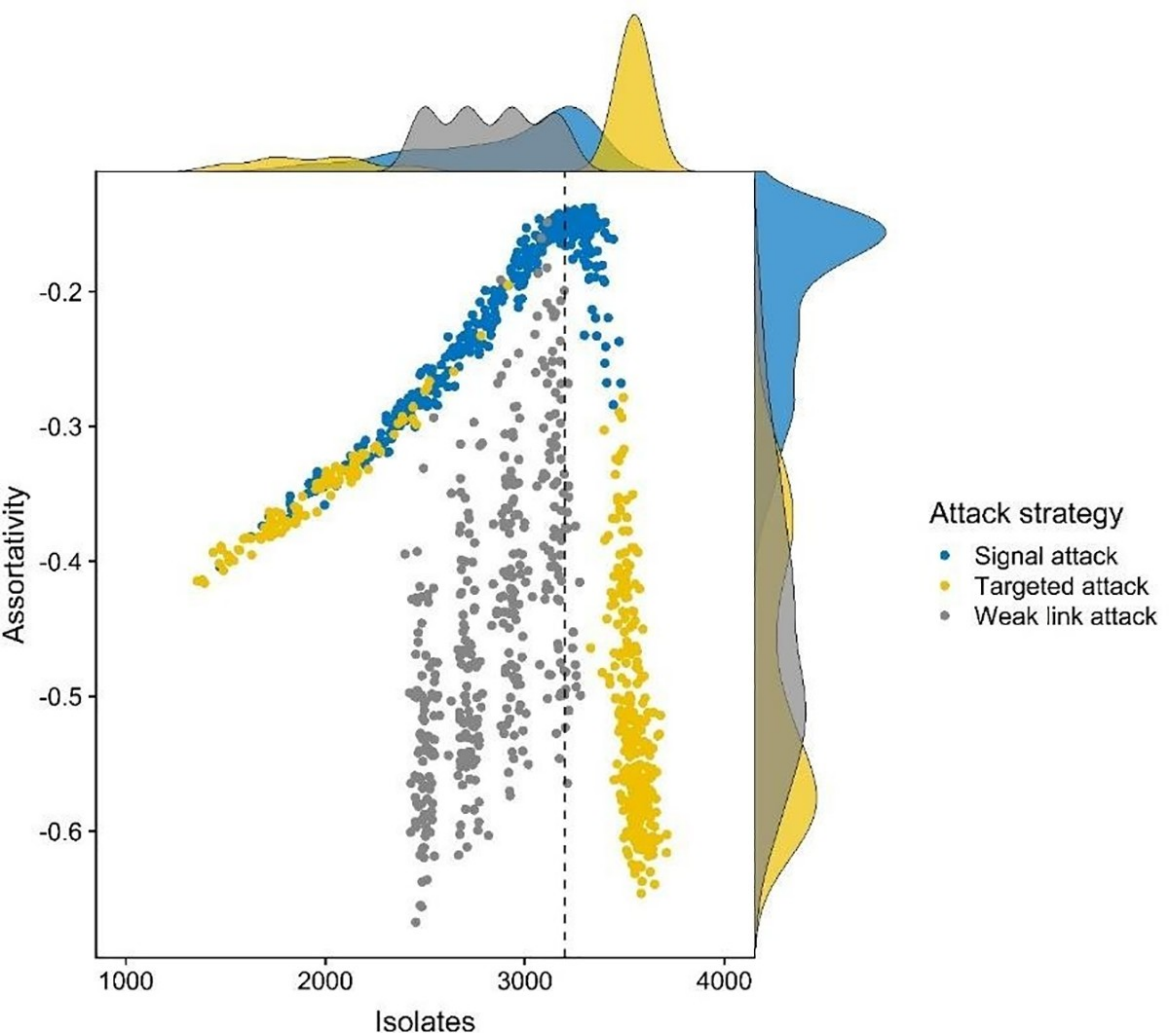
Association between assortativity and the number of isolates across treatment conditions (*n* = 1,200 networks). Density plots on the *x* and *y* axes denote the univariate distribution for assortativity and the number of isolates for each treatment condition. Dashed vertical line marks the approximate point at which the number of isolates is inversely associated with assortativity (3,200 isolates). Grey dots are targeted attacks (*n* = 400), yellow dots are weak link attacks (*n* = 400), blue dots are signal attacks (*n* = 400).

Our second concern is to identify how network behaviors change in the aftermath of an attack. On one hand, attacks may debilitate a network, leaving lasting damage and rendering it vulnerable to additional attacks; on the other hand, attacks may incentivize a network to insulate itself against further damage. We evaluate these possibilities by calculating the degree scaling coefficient and degree-degree correlation for each simulated network. Change in degree scaling is modest for each attack strategy (Fig 4A). Targeted and signal attacks tend to increase degree scaling, while weak link attacks tend to decrease it. This indicates that signal and targeted attacks increase network vulnerability, albeit modestly, by increasing the visibility of leading actors. Turning to degree-degree correlation (assortativity), targeted attacks do not yield noteworthy changes in assortativity at low levels of intervention, though higher levels of intervention do decrease assortativity. For weak link attacks, there is an inverse correlation between the level of intervention and assortativity (*r =* -.68, *P* < .001, Fig 4B), indicating that weak link attacks increase network vulnerability. Signal attacks tend to increase assortativity. However, these gains in assortativity stem, in part, from the large number of isolates which are generated by signal attacks (Fig 5, see S1 Appendix, Assortativity and Isolates for discussion). These results illustrate that intentional attacks increase network vulnerability in responsive networks, either by increasing preferential attachment or by decreasing degree-degree correlation.

Next, we assess the micro-mechanisms that generate these high levels of preferential attachment and low levels of degree-degree correlation. Results from our primary stochastic actor-oriented models suggest that much of the preferential attachment observed in the empirical network is driven by vendors’ reputations (cumulative sales ratings) (*β* = .0005, CI = [.0005.0005], *P* < .001, Model 1, S2 Table in S1 Appendix). To assess preferential attachment in the wake of an attack, we modeled vendors’ degree centrality in the simulated networks using linear mixed models, with simulated vendors nested in simulated networks, and simulated networks nested in empirically observed vendors. Consistent with stochastic actor-oriented models, vendor- reputations drive preferential attachment in the simulated networks (*β* = .162, CI = [.162, .162], *P* < .001, Model 3, S6 Table in S1 Appendix). This result replicates several prior findings on the importance of reputations for cooperation in economic games and criminal networks [6, 19, 37, 38]. Turning to the micro-level sources of degree-degree correlation, we model the difference in degree centralities between buyers and vendors who have exchanged drugs using linear mixed models, with differences in degree centralities nested in networks. As above, micro-level assortativity is largely driven by preferential attachment towards reputable vendors (*β* = .019, CI = [.019, .019], *P* < .001, Model 3, S7 Table in S1 Appendix), as well as the number of isolates in the network (*β* = -.001, CI = [-.001, -.001], *P* < .001, Model 3, S7 Table in S1 Appendix). This indicates that, when networks are attacked, buyers grow more cautious about their purchasing habits, leading them to simultaneously make fewer drug purchases and, when they do purchase, only purchase drugs from vendors with good reputations. In doing so, they increase overall network vulnerability.

## Discussion

The findings in this study provide important insight to the problem of network responsiveness. First, we find that cross-sectional methods can provide misleading assessments of network vulnerability when the network is dynamic. This suggests that prior evidence showing that social networks exhibit high levels of robustness [11] may need to be revisited with network responsiveness in mind. Second, we find that diffuse attack strategies can outperform targeted attacks in terms of curbing long-term network activity. This finding illustrates that attackers may be successful in debilitating network activity even when they have limited information on network structure or limited access to key players. This is consistent with prior studies showing that many networks are vulnerable to attacks conducted at random or with incomplete information on network structure [4, 5, 7]. Third, we find that most of the change in network activity and network structure after an attack can be attributed to the behaviors of weakly connected actors, rather than structurally integral ones. This is again in contrast to cross- sectional research on network vulnerability, which generally assumes that highly connected actors are a critical vulnerability in common network topologies [6–9].

Finally, results indicate that network attacks can create a game theoretic dilemma in responsive networks, where actors’ attempts to protect themselves by exchanging drugs with only the most reputable vendors ultimately increases network vulnerability. Consequently, collective action may be necessary to improve network robustness following intentional attacks, as egoistic action undermines network security. Future research on network responsiveness should consider how collective norms or rules for cooperation may promote network recovery and long-term robustness in disrupted environments.

Results also carry policy implications. Few prior studies have been able to tie levels of criminal activity to network-based interventions. Findings indicate that small-scale targeted attacks do little to curb drug trafficking in the long-run. This raises questions about crime policy recommendations based on cross-sectional research, which generally propounds allocating resources to identifying and arresting a handful of structurally integral criminals [3, 6, 9, 14, 18–22]. While findings for small-scale targeted attacks may be discouraging, results for diffuse attacks are enheartening. For one, we find that weak link attacks are, on average, the most effective for reducing levels of criminal activity. Moreover, one implication of the finding that weak link attacks decrease degree-degree correlation is that they may be effective for priming a network for future attacks. Likewise, results for signal attacks suggest that they may be a viable attack strategy when resources are unavailable to physically arrest market actors.

Our use of online drug trade data necessarily encounters limitations. In general, examining digital traces increases the reliability, accuracy, and scale of the data [39]. Moreover, our use of darknet drug trade data gives unique insight to a large, currently active, and dynamic criminal network. Nevertheless, it is an open empirical question whether results for crime conducted through a digital medium translate to the bulk of crime conducted offline. Likewise, a strength of agent-based simulation is the ability to conduct experiments at a scale which is typically unfeasible. A weakness, however, is that the data generated from simulation models are synthetic (albeit based on empirical data gathered from an active darknet drug market). If possible, data generated from experiments where attacks are carried out and change in the network is assessed would be ideal for validating our conclusions.

Despite the far-reaching scientific importance of network vulnerability, there has been markedly less research on the problem of network responsiveness [20, 21]. This omission is particularly pressing in research on social networks, where actors can be expected to exhibit rational adaptations to network disruptions. Results provide strong evidence that actors in criminal networks exhibit rational responses to attacks and that these adaptations can undermine overall network robustness. Moreover, findings reveal that cross-sectional methods can lead to misleading conclusions about network vulnerability when the network being studied is dynamic in nature. Collectively, results highlight the need to consider network responsiveness when examining the vulnerabilities of social and other dynamic networks.

## Materials and methods

Darknet drug markets are anonymous online marketplaces where users from across the globe can purchase illicit drugs, such as heroin and methamphetamine, from anonymous vendors and have the drugs delivered to their door steps through a postal service. They function akin to Clearnet markets (e.g., *eBay*), incorporating drug listings, vendor reputation scores, and histories of product reviews left by previous buyers. We constructed our network by gathering data from each vendor’s web page between the first date of market operation, January 2017, and February 2018. Additional details are provided in the S1 Appendix (Data). The Ohio State HSIRB exempt the study from ethical oversight as all data are digital trace data and can be accessed publicly. All data were analyzed using website specific pseudonyms (usernames) that are not connected in any known way to persons' true offline identities. No efforts were taken to anonymize data during analysis because darknet encryption software makes it extremely difficult to link usernames to individual persons.

Our agent-based simulation experiment was conducted in four steps. We discuss the design, assumptions, and mathematical definition of the agent-based simulation in detail in the S1 Appendix (Estimation). In Step 1, we fit a stochastic actor-oriented model to the observed network to identify the determinants of market growth [40]. The model entails simulating network change from the raw data and then fitting a multinomial logistic regression to the simulated data. Actors in the network are offered a probabilistically determined number of opportunities to change ties based on a rate function (S1 Appendix Estimation). When offered the opportunity to change a tie, an actor chooses the tie that offers the largest increase to the objective function:

$$f(\beta ,x)=\sum_{k}\beta_{k}s_{k}(x)$$

Where 𝛽_𝑘_ are the parameterized covariate effects provided by the researcher and (𝑥) are functions of the data computed on the observed network at each step in the simulation. An actor may choose not to form a tie if there is no tie which increases the value of the objective function. Tie changes are then recorded and the network state is updated. Since the simulation can be regarded as a continuous-time Markov chain, the actual passage of time between observations of the network is arbitrary [40]. As is common, we estimated the model through method of moments using a stochastic approximation algorithm. Additional details are provided in the S1 Appendix, along with a discussion of control variables, model specification, and full model results (S2 Table in S1 Appendix). After estimating the model and ensuring good fit, we recorded the parameter vector (𝛽) to be used in agent-based simulation.

In Step 2, we implemented the manipulations to the observed data as outlined in Table 1.

In Step 3, we initiated the agent-based simulation by using stochastic actor-oriented models to simulate a range of potential network outcomes based on the manipulated data. Since stochastic actor-oriented models have a simulation basis, this merely entailed estimating an agent-based model with the same rules for actor behavior as outlined above and storing the simulated networks [see 41, 42]. The parameters of the agent-based simulation are fixed to be equivalent to the coefficients estimated in Step 1 (Model 1, S2 Table in S1 Appendix). This ensures external validity by basing the parameters of the agent-based model on statistical analysis of empirical data [39, 40]. Since we estimate the agent-based model by using a stochastic actor-oriented model to simulate from manipulated data, the various attack strategies can be regarded as influencing agent behavior by changing their respective evaluations of the objective function (S1 Appendix, Simulation). We repeated the agent-based simulation 100 times for each condition to ensure that the results from the simulations are not idiosyncratic to a single stochastic process. In Step 4, we record structural characteristics of the networks and its actors for analysis. We recorded the number of ties, number of isolates, and actors’ degree centrality, and we computed the degree- degree correlation and degree scaling coefficient for the output networks. Equations for each structural measure are available in the S1 Appendix (Measurement). We analyzed the data using linear mixed models (random intercepts) for the degree centrality and difference in degree centrality outcomes, ordinary least squares regression for the drug trafficking volume outcome, and conditional logistic regression for the probability of becoming an isolate.

## Supporting information

S1 Appendix (DOCX)
