# Supplementary material for: The responsiveness of criminal networks to intentional attacks: Disrupting darknet drug trade
Source: PLoS One. 2020 Sep 10;15(9):e0238019. doi: 10.1371/journal.pone.0238019 (PMC7482914; doi:10.1371/journal.pone.0238019)
Supplement: S1 Appendix — (DOCX) [file pone.0238019.s001.docx]

Supplemental Information Appendix to “The Responsiveness of Criminal Networks to Intentional Attacks: Disrupting Darknet Drug Trade.”

This SI Appendix includes supplementary information on data collection procedures, analyses, and model validation. It also includes an extended discussion of the relationship between isolates and assortativity.

DATA

Our data were collected from *Silk Road 3.1. Silk Road 3.1* is currently one of the ten largest darknet markets in terms of overall trafficking. It is also a rare market which provides the full usernames of both buyers and vendors. This information is necessary to recreate the transaction network. *Silk Road 3.1* employs a mandatory review policy, which allows us to gather the entire population of transactions between buyers and vendors over the course of a year. These conditions have proven necessary to conduct network analysis on darknet markets in prior research (1 - 3).

We collected 14 months of network data from *Silk Road 3.1. Silk Road 3.1* is one of the largest and longest running darknet drug markets. Our data contains information on all drug transactions occurring in the market between January 2017 and February 2018. Prior to January 2017, *Silk Road 3.1* was *Silk Road 3.0*. After some website reconfiguration, the market rebooted itself and reset the transaction history for all buyers and vendors, providing a fresh start for the market. As such, our data reflects the entire population of drug transactions on *Silk Road 3.1* from January 2017 to February 2018*.*

We gathered our data from each vendor’s web page. Vendors’ web pages contain a listing of the products a vendor sells, a brief description of the vendor’s services (written by the vendor), and a list of product reviews for each transaction that a vendor has made. Sales reviews are mandatory on most darknet drug markets, including *Silk Road 3.1,* and are required to finalize a drug sale (though buyers can return and change their evaluation at any point in the future). Each product review contains the buyer’s numeric evaluation of the sale, the amount of money exchanged in a transaction, and the drug that the buyer purchased. We recorded information on vendors’ positive and negative sales ratings, the cost of the drugs a vendor sells, their geographic location (country level), and the types of drugs a vendor sells.

Since each product review reflects a transaction, we constructed the network of transactions between buyers and vendors based on these comments. The ties are equal to 1 if a transaction has occurred and equal to 0 otherwise. With binary measurement, we examine roughly 11,634 ties or unique drug transactions. Binary measurement is necessary to estimate stochastic actor-oriented models (discussed below). We treat the networks as bipartite, reflecting that ties are only possible between buyers and vendors.

ESTIMATION

We perform our analysis in four steps. In the first step we fit a stochastic actor-oriented model (SAOM) to our observed data. This allows us to ensure good model fit and to derive accurate parameter estimates for network growth. In the second step, we manipulate the data as per our treatment conditions. Third, we simulate a range of potential networks based on the manipulated data and the parameter weights identified in Step 1. Finally, we collect and analyze the output networks.

*Step 1. SAOM estimation*

Stochastic-actor oriented models (SAOM) are statistical models for longitudinal network data (4). Given multiple observations of a network, SAOM assumes that network change is a Markovian process, such that the differences between network states are formed through sequential and intermittent tie changes. Actors in the network are assumed to be able to control their outgoing ties based on a utility function, which is weighted by the parameterized covariate effects that the researcher provides. Change in the network is then simulated, where each actor is offered the opportunity to change a tie with another actor based on a rate function. The simulations are repeated many times to generate a probability distribution of network states from which model fit can be evaluated and parameter weights identified. Thus, SAOM can be most accurately conceptualized as an integration of agent-based modeling with classical methods for statistical inference.

SAOM is estimated as a series of simulated micro-steps. The SAOM begins by calculating a rate function for each actor at each period in the network. The rate function assigns the frequency with which actors manipulate their ties in a time period during the agent-based simulation. We estimate the model with a fixed-rate function, meaning that all actors have the same baseline probability of being offered the opportunity to change a tie:

$$\lambda_{i}=\rho$$

Where $\rho$ is the number of tie differences between temporally adjacent states of the network. The probability that an actor will be chosen to change a tie is

$$\frac{\lambda_{i}}{\sum\lambda_{i}}$$

Where $\sum\lambda_{i}$ is exponentially distributed.

Next, the simulation begins. Actors are probabilistically selected and offered the opportunity to manipulate a tie based on their rate function. Actors choose which tie to change based on an objective function:

$$f(\beta,x)=\sum_{k} \beta_{k}s_{k}(x)$$

Where $\beta_{k}$ are the parameterized covariate effects provided by the researcher and $s_{k}(x)$ are the network statistics computed on the observed network at each micro-step$.$The distinction between the raw data, *x,* and $s_{k}(x)$ is that $s_{k}(x)$ includes functions of the raw data that change during each micro-step; for instance, degree centrality. The tie change which offers the greatest increase in the objective function is the tie that forms (or dissolves) during a micro-step. If no tie change increases the value of the objective function, then an actor can also choose not to manipulate a tie. Since the actor forges one connection among many, the probability of tie formation is represented using a multinomial choice model,

$$\frac{exp(f(\beta,x))}{\sum exp(f(\beta,x`))}$$

Where the denominator represents the summation of the probability that an actor would choose any other alter than the one that they selected. Once an actor makes a decision about a tie change, the state of the network is updated to reflect their choice. Another actor is then selected based on their rate function, and the simulation proceeds to the next micro-step based on the new state of the network. The simulations are repeated many times to ensure that results are not idiosyncratic to a specific change process. Coefficients can be interpreted as the increase or decrease in log-odds that a buyer will purchase from a vendor given a one unit increase in the independent variable.

Model convergence is assessed by comparing network statistics simulated from the model to the observed final state of the network. A *t-*test is performed for each model parameter, where the null hypothesis is that there is no difference between the simulated statistics and the data. Smaller *t*-ratios indicate better fit. By convention, good convergence is obtained when the absolute value for parameter *t-*ratios is below .1, and the overall model *t*-ratio is below .25 (4).

We modeled change in the *Silk Road 3.1* network as a function of vendors’ reputations, affordability, country of origin, and history of transactions. We measured vendors’ reputations as vendors’ cumulative sales ratings (summation of all positive and negative sales ratings) at each wave. The measure is time varying and cumulative, where higher values indicate that a vendor has a history of good transaction reviews, and negative values indicate the opposite. To measure affordability, we constructed the mean transaction cost as the average cost of drug exchange with a vendor. While vendors’ listed prices are also available on their webpages, these prices are often unreliable since vendors may deliberately provide misleading quotes to attract buyers, and because costs may change over time. The countries of origin we specify are the US, Netherlands, Canada, and Germany. Additional countries were included in earlier models, such as Australia and France, but these variables were typically perfectly collinear with other model effects due to the small number of vendors located in these countries (e.g., < 3). Finally, we also control for vendors’ degree centrality in supplemental models to provide a second indicator of vendor reputations, where higher values indicate vendors have engaged in more drug exchanges (Model S2, Table S2, below).

Given the complexity of SAOM, there are a few statistical issues which warrant attention. The first important issue is time heterogeneity, where the effects of some coefficients vary across waves. Time heterogeneity can problematize SAOM convergence. We take a few steps to limit the influence of time heterogeneity. First, one reason for the issue is that the market was shut down for a month in July 2017 to undergo maintenance. Thus, there were no transactions in July 2017 and the market took roughly two months to recover its transaction volume. The recommended approach to handling time heterogeneity is to combine multiple waves of data

Table S1. Descriptive statistics for 3 Waves of *Silk Road 3.1* drug transaction data.

|  | Wave 1 | Wave 2 | Wave 3 | Aggregate |
| --- | --- | --- | --- | --- |
| Density | .026 | .013 | .021 | .014 |
| Degree scaling coefficient | 2.10 | 1.52 | 1.46 | 1.52 |
| Degree-degree correlation | -.07 | -.07 | -.07 | .05 |
| Number of transactions | 1,110 | 6,736 | 9,001 | 16,847 |
| Number of vendors | 50 | 121 | 101 | 169 |
| Number of buyers | 505 | 2,977 | 4,323 | 7,126 |
| Vendor indegree (mean/SD) | 6.57 (24.19) | 39.86 (79.45) | 53.35 (118.02) | 99.68 (195.14) |
| Buyer outdegree (mean/SD) | .156 (.816) | .945 (1.818) | 1.26 (1.98) | 2.36 (2.66) |
| Transaction cost (mean/SD) | 570.48 (1,013.69) | 351.18 (702.29) | 120.40 (326.63) | 242.61 (585.10) |
| Vendor reputations (mean/SD) | 102.78 (205.27) | 309.71 (546.03) | 471.54 (951.45) | 471.54 (951.45) |
| *Vendor country* |  |  |  |  |
| US | 44% | 35% | 42.6% | 39.1% |
| Netherlands | 8% | 13.3% | 10.9% | 11.8% |
| Germany | 2% | 2.5% | 0% | 1.8% |
| Canada | 2% | 2.5% | 2.9% | 2.4% |
| Other | 44% | 46.3% | 43.6% | 46.9% |

(5). Thus, we aggregate our data to three waves where each wave covers a four or five-month window. Wave 1 represents drug exchanges between January 2017 and April 2017, Wave 2 represents drug exchanges between May 2017 and September 2017, and Wave 3 represents drug exchange between October 2017 and February 2018. Such data reduction has little consequence for the statistical properties of a SAOM, since the actual passage of time between observations of a network are arbitrary (4). We present descriptive statistics for the network at each wave in Table S1. Wave 3, therefore, acts as our dependent variable, and the simulated output networks from our agent-based simulation (discussed below) are the model’s estimates of the Wave 3 network. The second strategy for handling time heterogeneity is to include dummy variables for each time period. Since we observe two time periods (change between Wave 1 and 2, change between Wave 2 and 3), we include a dummy variable equal to 1 in the Wave 2 – 3 change period and equal to zero in the Wave 1 – 2 change period.

The second issue that can arise in SAOM is time-varying network composition. SAOM requires that each observation of a network contains the same number of actors for estimation. Since the number of actors changes per wave, we include an offset parameter to constrain the pool of potential alters per wave. The offset parameter is a time varying dyadic covariate represented as an *n* x *m* affiliation matrix, where *n* is the number of buyers and *m* is the number of vendors. The matrix contains structural zeroes for all cells where a tie can possibly form (e.g., where both the buyer and the vendor are present in the market at that timepoint) and ones for all cells where a tie is impossible (where either the vendor or buyer is not yet present in the market). This is the conventional approach to handling unbalanced network panel data in longitudinal network analysis (5, 6).

The third issue is collinearity. Since vendors’ cumulative sales ratings are defined by the number of sales a vendor has made, vendors’ reputations are highly correlated with their degree centrality (*r* = .988, *P <*.001 at Wave 2, *r* =.996, *P* <.001, at Wave 1). Consequently, the two measures are collinear when included in the model simultaneously. Below, we provide two sets of models, one with vendors’ cumulative sales ratings and the other with degree centrality. Results are substantively consistent across models.

We estimate the model using method of moments and a stochastic approximation algorithm to identify the moment estimator (7). To ensure good model fit, we repeat estimations until the absolute value for all *t*-convergence ratios for all model parameters is below .1 and the overall model convergence ratio has an absolute value less than .25.

Table S2 presents results from SAOM. Model 1 includes vendors’ reputations and excludes degree centrality. The rate parameter for the second change period is larger than the first, reflecting that buyers have more opportunities to engage in drug exchanges between the second and third waves than between the first and second waves (consistent with Table S1). The negative coefficient for buyers’ outdegree indicates that the baseline probability that a buyer will purchase from a vendor is quite small. The positive coefficient for vendors’ reputations indicates that a one unit increase in vendors’ reputations is associated with a .05% ($e^{.0005}$) increase in the odds that a buyer will purchase from that vendor. Operating over a range of -5 to 8,058, this effect explains substantial variation in tie formation between waves. The positive value for mean transaction costs indicates that buyers are willing to pay a premium to purchase quality drugs. The coefficients for country indicate that vendors in the US and Germany are less appealing than others, and the positive coefficients for Canada and the Netherlands indicate that vendors in these countries are more appealing. The negative coefficient for the offset matrix reflects the true-by-construction fact that buyers and vendors who are not on the market have lower odds of engaging in drug exchange. The period 2 – 3 time dummy indicates that, after controlling for other characteristics, buyers have a lower probability of purchasing between Waves 2 and 3 than between Waves 1 and 2. Model 2 excludes vendors’ reputations and includes degree centrality. Results are consistent with Model 1. The positive coefficient for vendors’ degree centrality indicates that buyers are more likely to purchase from vendors who have engaged in large amounts of drug exchange in the past.

| Independent Variables | Model 1 | |  | Model 2 | |
| --- | --- | --- | --- | --- | --- |
|  | β (SE) | *t-*ratios |  | β (SE) | *t-*ratios |
| Rate (period 1) | 2.8678*** (.1401) | -.0126 |  | 2.8616***  (.1356) | .0015 |
| Rate (period 2) | 9.9636*** (.3495) | -.0299 |  | 10.6191***  (.3510) | .0141 |
| Buyer degree centrality  (density) | -3.5040*** (.0166) | -.0166 |  | -3.5060***  (.0161) | .0190 |
| Vendor reputation | .0005***  (.0001) | .0160 |  | - | - |
| Vendor degree centrality | - | - |  | .0053***  (.0001) | .0092 |
| Mean transaction cost | .0001***  (.0000) | -.0104 |  | .0001***  (.0000) | .0184 |
| *Vendor country* |  |  |  |  |  |
| US | -.1419***  (.0211) | .0010 |  | -.0674***  (.0210) | .0004 |
| Netherlands | .4129***  (.0219) | .0019 |  | .2721***  (.0232) | .0023 |
| Germany | -1.2202***  (.1317) | -.0100 |  | -1.1830***  (.1375) | -.0012 |
| Canada | .4436***  (.0331) | -.0026 |  | .3235***  (.0330) | -.0029 |
| Offset matrix | -1.0672***  (.0225) | .0071 |  | -1.0170***  (.0227) | -.0049 |
| Period 2 - 3 (dummy) | -.2213***  (.0286) | .0122 |  | -.3001***  (.0272) | .0064 |
| Model *t* ratio | - | .1072 |  |  | .0374 |

Table S2. Results from SAOM analysis on 3 Waves of *Silk Road 3.1* drug transaction data. ****p*<.001; 7,126 buyers, 169 vendors, 11,634 ties. Number of phase 2 subphases = 6; Number of phase 3 simulations = 10,000.

*Step 2. Manipulation*

In the manipulation step, we manipulate the data to correspond to different network attack strategies. Since our network is dynamic, it is crucial to consider at what time point interventions are most meaningful. Our SAOM treats the Wave 3 network as the dependent variable, and thus the simulated output networks are the model’s best estimate of change between Waves 2 and 3. For this reason, we implemented each attack strategy at Wave 2. For targeted attacks, we first sorted vendors by their degree centrality at Wave 2 and then deleted those in the top 20^th^, 40^th^, 60^th^, and 80^th^ percentile, reflecting low, medium-low, medium-high, and high levels of intervention. For weak link attacks, we sorted buyers by their degree centrality at Wave 2, and then deleted those in the bottom 20^th^, 40^th^, 60^th^, and 80^th^ percentile. For signal attacks, we reduced vendors’ reputations at Wave 2 by 20%, 40%, 60%, and 80%, again reflecting low, medium-low, medium-high, and high levels of intervention.

*Step 3. Simulation*

In the simulation step, we simulate network change from the manipulated data. Given the simulation basis for SAOM, Snijders and Steglich (8) have recently observed that SAOMs can be used as a general framework for agent-based modeling. First, a researcher specifies a parameter set to a SAOM. Second, the researcher provides input data. Third, the researcher initiates an agent-based simulation using the same rules for agent-behavior as in a SAOM. However, instead of estimating parameters from the data, the researcher fixes the parameter set to reflect meaningful values. Fourth, the researcher initiates the agent-based simulation portion of a SAOM with fixed parameter weights. This results in a set of outcome networks which reflect potential network states that could form given the data-generating process specified in the parameter set. In the case of a simulation experiment, raw data can also be manipulated to identify treatment effects based on hypothetical network interventions (9, 10).

In our study, we fix the parameter weights to be equal to those obtained in Step 1. We then use SAOM to simulate a series of outcome networks for each manipulated data set. By doing so we are able to assess how a darknet drug market would change following a targeted, weak link, or signal attack. The benefit of this approach is to use a well-established modeling framework to provide the parameters for an agent-based model. Using estimates from SAOM on empirical data and then using SAOM for simulation thus ensures that the agent-based model has high external validity, as the parameters of the agent-based model are obtained from statistical analysis of empirical data. This is a boon to researchers since model validation is perhaps the biggest concern in drawing conclusions about the social world from agent-based modeling (11 - 13). Based on a SAOM framework, our agent-based model makes the following assumptions:

1. Buyers control their purchases (outgoing ties).
2. Buyers make decisions about purchasing based on the perceived utility of those purchases.
3. Decisions about from whom to purchase are mutually exclusive.
4. The underlying time parameter is continuous.
5. Network change is sequential; no two transactions occur simultaneously.
6. The final state of the network is the outcome of a Markov process.

*Step 4. Measurement*

In Step 4, we record the structural characteristics of the simulated output networks for statistical analysis. The first is the number of ties (unique drug transactions):

$$\sum x_{ij}$$

Where *x* takes a value of 1 if *i* has purchased drugs from *j* and is equal to zero otherwise. The second measure is the number of isolates:

$$\sum I(k_{i}=0)$$

Where $I(k_{i}=0)$ takes a value of 1 if *i* has a degree centrality of zero and is equal to zero otherwise. The third measure is the degree scaling coefficient, equal to the parameter of a power-law distribution (14). We identified the parameter using the Kolmogorov-Smirnov method as per Clauset, Shalizi, and Newman (15). This entails minimizing the distance between the cumulative distribution function for the degree distribution ($P_{deg}(\kappa)$) and the CDF for the power-law ($P_{\gamma}(\kappa))$:

$$\hat{\gamma}={arg min}_{\gamma}(max|P_{deg}(\kappa)-P_{\gamma}(\kappa)|)$$

Where *γ* is the degree-scaling coefficient and $\hat{\gamma}$is its expectation. The final measure is assortativity, which is the Pearson correlation for the degree distribution:

$$\frac{\sum_{zk} zk(e_{zk}-q_{z}q_{k})}{\sigma_{q}^{2}}$$

Where *z* and *k* are the degree centralities of two actors, $e_{zk}$ is the joint probability distribution of the two degree centralities of two actors connected by tie, $q_{z}$ and $q_{k}$ are the marginal probability distributions of the two degree centralities of two actors (excluding the tie connecting the actors), and the denominator is the variance of the distribution for *q* (16)*.*

ASSUMPTIONS

Our agent-based model makes the assumption of bounded rationality. Agents are assumed to optimize a utility function based on their subjective evaluations of short-term possible actions. While the assumption of rationality has been critiqued in the social sciences, we believe it is appropriate in the current context for several reasons. First, a well-developed stream of criminological theory regards offenders as rational actors (17 - 19). Offenders make evaluative decisions about offending opportunities by weighing the risk of punishment against the situational gains accrued from offending decisions. While the applicability of this theoretical tradition to crimes of passion is disputable, economic transactions—especially in drug markets—are typically more closely informed by rational choice behavior (20). Second, online drug exchange promotes evaluative decision making. Users must first download Tor software, access the market, convert currency into Bitcoin, navigate the website, and finally purchase a drug before waiting several days to receive it. Online drug purchasing thus imposes delayed gratification, which implies deliberative choice behavior. This elongated process contrasts with the geographic context of open-air drug exchange, where users may purchase drugs on a whim.

Finally, some assumption of rationality is usually imposed on agent-based models of social systems because, if agents are not assigned some form of systematic motivation, it is difficult to examine how they interact in social space (11, 12). We believe that our rational choice assumption is in fact *more flexible* than the choice assumptions made in most agent-based models of social systems because the utility function we derive follows from a generalized linear modeling framework (see Equation 3 for the objective function) and the parameters we assign are estimated from the data. This aligns more closely with the real-world dynamics of social systems, which are wrought with noisy signals, imperfect information, and randomness.

MODEL VALIDATION

A large concern in the literature on agent-based modeling is ensuring that the simulation represents an empirically meaningful change process (12, 13). A strength of using SAOM as an agent-based model is that the parameters of the simulation can be empirically validated on the observed data (8 - 10). The small *t*-ratios for our empirical model and all parameters reflect strong model convergence and good fit, indicating high external validity. A common approach for ensuring the agent-based model results are not idiosyncratic is to replicate the agent-based model multiple times and aggregate over the results (11 - 13). This ensures that the results from a single agent-based model are not unduly affected by the starting seed value of the random number generator or other stochastic processes underlying the model. By reiterating the agent-based model 100 times, we confirm the robustness of our results across a range of potential starting seeds.

DESCRIPTION OF CONTROL CONDITION

S1 Fig presents descriptive statistics for the simulated output networks in the control condition. The mean transaction volume is 6,863.13 with a standard deviation of 741.81 and a range of 4,879 to 8098. The mean degree scaling coefficient (γ) is 1.65 with a standard deviation of .07 and a range of 1.43 to 1.81, reflecting a right-skewed degree distribution which is slightly below the standard threshold for a scale-free network (14). The mean assortativity is -.33, reflecting a tendency towards disassortativity (16), with a standard deviation of .05 and a range of -.42 to -.18. The mean number of isolates is 2,055.93, reflecting that roughly 28% of the network are isolates, with a standard deviation of 364.75 and a range of 1,177 to 3,055.


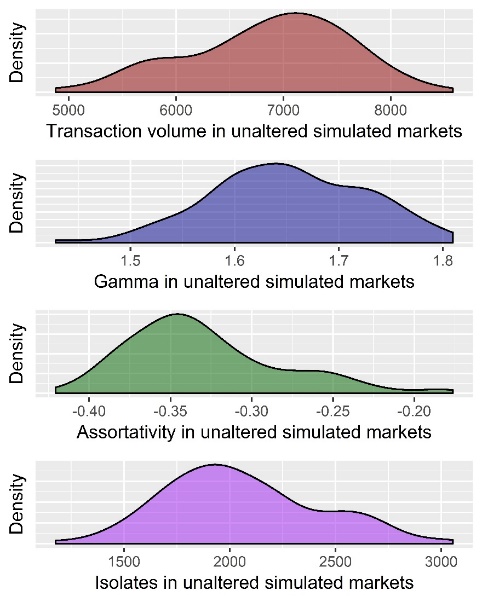


S1 Fig. Characteristics of the Control Condition.


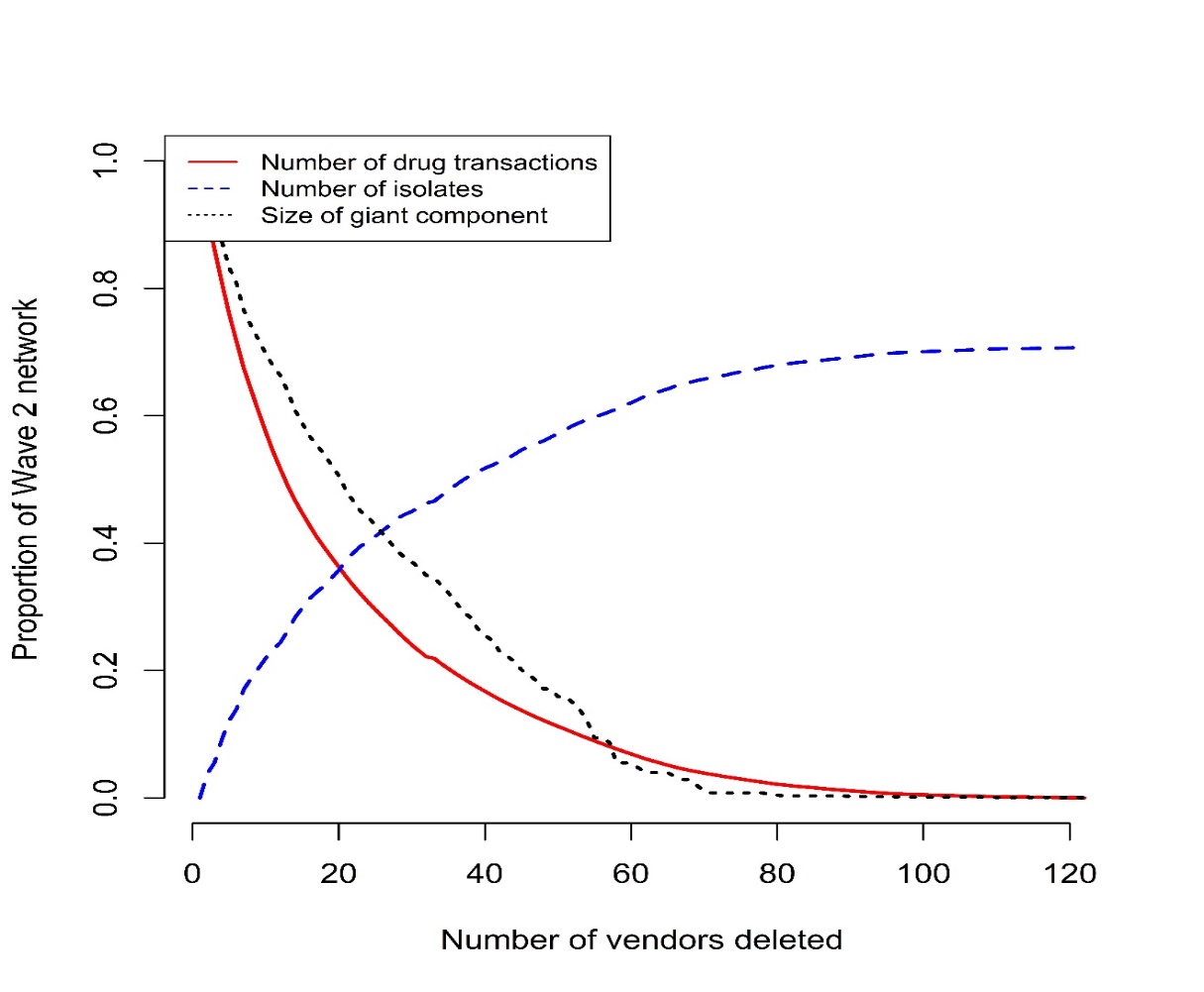


S2 Fig. Nodal deletion simulations of *Silk Road 3.1* network at Wave 2. *X* axis is the number of vendors deleted, *y* axis is the proportion of the Wave 2 network. Red line tracks the change in the number of drug transactions, the blue line tracks the change in the number of isolates, and the black line tracks the change in the size of the giant component.

CROSS-SECTIONAL NETWORK VULNERABILITY

We assessed cross-sectional network vulnerability using nodal deletion simulations at Wave 2—the moment where the network is attacked. At each step, we sorted vendors by their degree centrality and then deleted the vendor with the largest degree centrality. We repeated this until the network was completely dismantled (121 deletions). S2 Fig plots results from nodal deletion simulations. The network is entirely dismantled after 121 deletions but is functionally debilitated well before that. For instance, by the time 50 vendors are deleted, fewer than 10% of the original ties remain in the network, the largest component is 15.8% of its starting size, and 77.8% of all actors are rendered isolates. By the time 10 vendors are deleted, the giant

|  | 1 | 2 |  |
| --- | --- | --- | --- |
| Weak link attack ^a^ | -3.919^***^ | -2.404^***^ |  |
|  | (.106) | (.089) |  |
| Weak link attack ^c^ |  |  |  |
| Targeted attack ^a^ | -2.125^***^ | -.611^***^ |  |
|  | (.106) | (.089) |  |
| Signal attack ^a^ | -1.665^***^ | -.151 |  |
|  | (.106) | (.089) |  |
| Level of intervention |  | -.606^***^ |  |
|  |  | (.0177) |  |
|  |  |  |  |
| Intercept | 6.863^***^ | 7.469^***^ |  |
|  | (.095) | (.071) |  |
|  |  |  |  |
| F | 1,618^***^ | 1,216^***^ |  |
| R^2^ | .60 | .79 |  |
|  | | | |

Table S3. OLS Models of the number of drug transactions in a network, *n* = 1,300. ****p*<.001. Two-tailed tests. Unstandardized regression coefficients and standard errors for the number of isolates and number of components are divided by 1,000 in all models. ^a^ Control group is referent.

component is 69.8% of its original size, 57.5% of the original ties remain, and 29.8% of all actors are rendered isolates. Collectively results from nodal deletion simulations suggest that the *Silk Road 3.1* network is vulnerable to intentional attacks at Wave 2.

CHANGE IN DRUG TRAFFICKING VOLUME

We consider here which attack strategy has the greatest average effect on levels of drug exchange. We model variation in the number of drug transactions in a network using ordinary least squares regression. Model 1 in Table S3 includes the attack strategy. Compared to the control group, weak link attacks prevent, on average, 3,919 more drug transactions, targeted attacks prevent 2,125 drug transactions, and signal attacks prevent 1,665 drug transactions. The R^2^ is .60, indicating that 60% of the variance in the level of drug trafficking on the network can be explained by the attack strategy. Model 2 includes the level of intervention. For each increase in the level of intervention, an additional 606 drug transactions are prevented. The coefficients for attack strategy decline substantially, though the rank order remains the same. Signal attacks are not a significant predictor of the level of drug transactions in Model 2. The R^2^ for Model 2 is .79, indicating that 79% of the variation in levels of drug trafficking can be explained by accounting for network attacks and levels of intervention.

|  | |  |  |  |  |  |  |  |
| --- | --- | --- | --- | --- | --- | --- | --- | --- |
|  |  | | | |  |  |  |  |
|  | |  | 1 | 2 | 3 |  |  |  |
| Vendor^a^ | |  | -3.629^***^ | -3.448^***^ | -3.412^***^ |  |  |  |
|  | |  | (.011) | (.014) | (.016) |  |  |  |
| Weak buyer^a^ | |  | .258^***^ | .464^***^ | .529^***^ |  |  |  |
|  | |  | (.057) | (.049) | (.050) |  |  |  |
| Targeted attack ^b^ | |  | - | .228^***^ | -.006 |  |  |  |
|  | |  | - | (.017) | (.013) |  |  |  |
| Weak link attack ^b^ | |  | - | .367^***^ | .148^***^ |  |  |  |
|  | |  | - | (.017) | (.002) |  |  |  |
| Signal attack ^b^ | |  |  | .134^***^ | -.091^***^ |  |  |  |
|  | |  |  | (.002) | (.014) |  |  |  |
| Level of intervention (continuous) | |  | - | - | .091^***^ |  |  |  |
|  | |  | - |  | (.004) |  |  |  |
|  | |  |  |  |  |  |  |  |
| χ^2^ | |  | 134,566^***^ | 141,963^***^ | 148,562^***^ |  |  |  |
| AIC | |  | 6.12 x 10^5^ | 6.12 x 10^5^ | 6.12 x 10^5^ |  |  |  |
| BIC | |  | 6.12 x 10^5^ | 6.12 x 10^5^ | 6.12 x 10^5^ |  |  |  |

Table S4. Conditional logistic regression of the probability of becoming an isolate, *n* = 10,306,339, 7,295 strata, 1,300 clusters. ****p*<.001. Two-tailed tests. Unstandardized regression coefficients are log-odds ratios. Standard errors are clustered in networks; actors are strata. Model estimated using the Breslow method. ^a^Reference category is buyers with degree centrality of two or more in Models 1 and 2. ^b^ Reference category is the control group.

PROBABILITY OF BECOMING AN ISOLATE

To assess the probability of becoming an isolate, we estimated a series of conditional logistic regressions, with standard errors clustered on networks. Since our simulations contain 7,295 actors across 13 levels of the experiment, actors are, themselves, matched pairs. To correct, we treat actors as strata. Of primary interest is the number of weakly connected actors. We measure this with a dummy variable, equal to 1 if a buyer has a degree centrality of 1 or zero in Wave 2, and equal to zero otherwise. We also control for whether an actor is a vendor. The reference category for both of these variables are buyers whose degree centrality is 2 or greater at Wave 2.

Table S4 presents results. Results illustrate that weakly connected buyers are significantly more likely to become isolates, while vendors are significantly less likely to become isolates compared to active buyers. Targeted attacks do not significantly affect the probability of an actor becoming an isolate after controlling for the level of intervention (Model 3), while weak link

attacks increase the probability of becoming an isolate and signal attacks decrease it. While this

latter result is counterintuitive, it merely reflects that most of the effect of signal attacks comes

from its interaction with the level of intervention (e.g., Figs 2 and 4, main text). Intuitively, as

the level of intervention increases, so does the probability of becoming an isolate. Table S5

|  |  |  |  |  |  |  |
| --- | --- | --- | --- | --- | --- | --- |
|  | | |  |  |  |  |
|  | 1 | 2 | 3 |  |  |  |
| Vendor - strong buyer | -3.629^***^ | -3.448^***^ | -3.412^***^ |  |  |  |
|  | (.011) | (.014) | (.015) |  |  |  |
|  |  |  |  |  |  |  |
| Weak buyer – strong buyer | .258^***^ | .464^***^ | .529^***^ |  |  |  |
|  | (.049) | (.049) | (.050) |  |  |  |
|  |  |  |  |  |  |  |
| Weak buyer - vendor | 3.887^***^ | 3.912^***^ | 3.941^***^ |  |  |  |
|  | (.045) | (.041) | (.041) |  |  |  |
|  | | |  |  |  |  |

Table S5. Tukey pairwise rank comparisons from conditional logistic regression in Table S3, *n* = 10,306,339. ****p*<.001. Two-tailed tests. Unstandardized regression coefficients are log-odds ratios. Standard errors are clustered in networks. Model numbers correspond to models in Table S4.

shows results for Tukey pairwise rank comparisons, which indicate that weakly connected buyers are also significantly more likely to become isolates than vendors.

CHANGE IN PREFERENTIAL ATTACHMENT

To address the micro-mechanisms that drive preferential attachment in the wake of an attack, we use three-level mixed models to predict vendors’ degree centrality in the simulated networks, with vendor-network combinations nested in networks, and networks nested in empirically observed vendors (*n* =169). We nest networks in empirically observed vendors because each vendor is observed in every network (unless they have been deleted). We control for vendors’ mean transaction cost at Wave 2, vendors’ Wave 2 degree centrality, vendors’ Wave 2 cumulative sales ratings (reputation), the attack strategy, and level of intervention. Due to the large data in these analyses (*n* = 255,204), we were able to detect meaningful effects for reputation and degree centrality despite high correlations.

Results indicate that reputation drives much preferential attachment: a one unit increase in reputation is associated with a .097 increase in vendors’ degree centrality. This is consistent with stochastic actor-oriented models (Table S2). Affordable prices are also a determinant of preferential attachment, though the effect is much weaker. Vendors’ Wave 2 degree centrality is negatively associated with vendors’ degree centrality in the simulated networks. This reflects that buyers are dissuaded from purchasing from highly connected vendors if those vendors have no reputation to speak of—that is, when reputation is held constant. Signal attacks and targeted attacks tend to increase vendors’ degree centrality, while weak link attacks decrease it. This is consistent with Fig 4*A.* Higher levels of intervention also decrease preferential attachment after controlling for attack strategy, reflecting that there are fewer drug transactions in networks which have been attacked aggressively (higher levels of intervention).

|  | | |  |  |  |  |  |  |  |  |
| --- | --- | --- | --- | --- | --- | --- | --- | --- | --- | --- |
|  |  |  | | |  |  |  |  |  |  |
|  | | | 1 | 2 | 3 |  |  |  |  |  |
| Reputation | | | .160^***^ | .162^***^ | .162^***^ |  |  |  |  |  |
|  | | | (.003) | (.003) | (.003) |  |  |  |  |  |
| Price | | | -.012^**^ | -.017^**^ | -.017^**^ |  |  |  |  |  |
|  | | | (.003) | (.002) | (.002) |  |  |  |  |  |
| Degree centrality (Wave 2) | | | -.120^***^ | -.126^***^ | -.126^***^ |  |  |  |  |  |
|  | | | (.006) | (.006) | (.006) |  |  |  |  |  |
| Targeted attack ^a^ | | |  | -.233 | 4.454^***^ |  |  |  |  |  |
|  | | |  | (.755) | (.992) |  |  |  |  |  |
| Signal attack ^a^ | | | - | -4.237^***^ | .543^***^ |  |  |  |  |  |
|  | | |  | (.747) | (.994) |  |  |  |  |  |
| Weak link attack ^a^ | | | - | -8.184^***^ | -3.884^***^ |  |  |  |  |  |
|  | | |  | (.884) | (1.065) |  |  |  |  |  |
| Level of intervention (continuous) | | | - | - | -1.909^***^ |  |  |  |  |  |
|  | | |  |  | (.262) |  |  |  |  |  |
| Intercept | | | 18.539^***^ | 23.755^***^ | 26.149^***^ |  |  |  |  |  |
|  | | | (.440) | (.766) | (.834) |  |  |  |  |  |
| Variance component (networks) | | | .169 | .000 | .000 |  |  |  |  |  |
|  | | |  |  |  |  |  |  |  |  |
| Variance component (vendors) | | | 2,577 | 2,578 | 2,580 |  |  |  |  |  |
|  | | |  |  |  |  |  |  |  |  |
| AIC | | | 3.22 x 10^6^ | 3.22 x 10^6^ | 3.22 x 10^6^ |  |  |  |  |  |
| BIC | | | 3.22 x 10^6^ | 3.22 x 10^6^ | 3.22 x 10^6^ |  |  |  |  |  |

Table S6. Three-level linear mixed model of vendors’ degree centrality, *n* = 255,204 for simulated vendors, *n* = 1,300 for networks, *n* = 169 for unique vendors. ****p*<.001. Two-tailed tests. Unstandardized regression coefficients and standard errors reported. Model estimated using maximum likelihood. ^a^Reference category is the control group.

MICRO-MECHANISMS DRIVING DEGREE-DEGREE CORRELATION

To assess the micro-level sources of assortativity, we examined differences in buyers’ and vendors’ degree centrality. Recall that assortativity refers to the difference in degree centralities between two actors connected by a tie. To model this at the micro-level, we reconstructed the edge list for each network, where each observation is a tie in the network. We then assigned weights to each tie equal to the difference in degree centralities between the two actors

incident to the tie. In these regards, the model can be conceptualized as nesting ties within

|  | |  |  |  |  |  |  |  |  |
| --- | --- | --- | --- | --- | --- | --- | --- | --- | --- |
|  |  |  | |  |  |  |  |  |  |
|  | | 1 | 2 | 3 |  |  |  |  |  |
| Reputation | | .019^***^ | .019^***^ | .019^***^ |  |  |  |  |  |
|  | | (.000) | (.000) | (.000) |  |  |  |  |  |
| Price | | .000^***^ | .000^***^ | .000^***^ |  |  |  |  |  |
|  | | (.000) | (.000) | (.000) |  |  |  |  |  |
| Degree centrality (Wave 2) | | -.003^***^ | -.003^***^ | -.003^***^ |  |  |  |  |  |
|  | | (.000) | (.000) | (.000) |  |  |  |  |  |
| Number of isolates | | -.001^***^ | -.001^***^ | -.001^***^ |  |  |  |  |  |
|  | | (.000) | (.000) | (.000) |  |  |  |  |  |
| Signal attack ^a^ | | - | -.359^***^ | -.234^***^ |  |  |  |  |  |
|  | |  | (.018) | (.023) |  |  |  |  |  |
| Targeted attack ^a^ | | - | .406^***^ | .522^***^ |  |  |  |  |  |
|  | | - | (.193) | (.228) |  |  |  |  |  |
| Weak link attack ^a^ | | - | .552^***^ | .425^***^ |  |  |  |  |  |
|  | | - | (.018) | (.023) |  |  |  |  |  |
| Level of intervention (continuous) | | - | - | -.056^***^ |  |  |  |  |  |
|  | |  |  | (.006) |  |  |  |  |  |
| Intercept | | 2.787^***^ | 3.189^***^ | 3.161^***^ |  |  |  |  |  |
|  | | (.559) | (.030) | (.030) |  |  |  |  |  |
| Variance component (networks) | | 192,437 | 54,056 | 51,330 |  |  |  |  |  |
| AIC | | 1.15 x 10^8^ | 1.15 x 10^8^ | 1.15 x 10^8^ |  |  |  |  |  |
| BIC | | 1.15 x 10^8^ | 1.15 x 10^8^ | 1.15 x 10^8^ |  |  |  |  |  |

Table S7. Two-level linear mixed model of absolute differences in degree centralities, *n* = 7,060,303 for ties, *n* = 1,300 for networks. ****p*<.001. Two-tailed tests. Unstandardized regression coefficients and standard errors divided by 1,000. Model estimated using pseudolikelihood. ^a^Reference category is the control group.

networks. We model the difference in degree centralities using two-level linear mixed models, with ties nested within networks. We control for vendors’ reputation, mean transaction cost, and Wave 2 degree centrality. Due to the large data in these analyses (*n* = 7,060,303), we were able to detect meaningful effects for reputation and degree centrality despite high correlations. We also controlled for the level of intervention, the attack strategy, and the number of isolates in the network.

Table S7 presents results from linear mixed models. The reputation coefficient is positive, reflecting the absolute difference in degree centralities tends to be larger when buyers purchase from reputable vendors (consistent with Table S6 and Table S2). The mean transaction cost coefficient is significant, but the coefficient is zero, reflecting a very small effect. Vendors’ Wave 2 degree centrality is negative (consistent with Table S6), but the effect is also small. Differences in degree centralities tend to increase more-so after a targeted attack than after a signal attack, while signal attacks tend to increase differences in degree centralities more-so than weak link attacks. This latter result is likely due to the large numbers of actors which are deleted by weak link attacks. The number of isolates is negatively associated with differences in degree centrality, reflecting that the number of isolates increases assortativity (discussed below). The level of intervention is negatively associated with differences in degree centrality after controlling for attack strategy. Consistent with results reported in the main text, signal attacks tend to decrease differences in degree centralities (increase degree-degree correlation), while targeted and weak link attacks tend to increase them (decrease degree-degree correlation). Results align with results above, illustrating that preferential attachment to reputable vendors largely drives the declines in degree-degree correlation following an attack.

ASSORTATIVITY AND ISOLATES

The purpose of this section is elaborate on the positive association between change in the number of isolates and change in assortativity. Assortativity is measured as the degree-degree correlation in a network. Assuming an undirected network, it is computed as the Pearson’s product moment correlation coefficient of the degree distribution (16),

$$r=\frac{\sum_{jk} jk(e_{jk}-q_{j}q_{k})}{\sigma_{q}^{2}}$$

Where *j* and *k* are the degree centralities of two vertices, $e_{jk}$ is the joint probability distribution of the two degree centralities of two vertices connected by an edge, $q_{j}$ and $q_{k}$ are the marginal probability distributions of the two degree centralities of two vertices (excluding the edge connecting the two actors), and the denominator is the variance of the distribution for *q.* Note here that the function assumes the existence of an edge connecting two vertices. Thus, by design, isolates are excluded.

Now consider a symmetric and binary one-mode network *A* with an arbitrary number of actors, where $r_{A}$ is its assortativity. Suppose a single actor *i* could either have a degree centrality of 1 or be an isolate. Since *k =* 1 is the smallest obtainable degree centrality in a binary network that will be included in calculations of assortativity, $r_{A}$ will be smaller if *i* has a degree centrality of 1 than if *i* is an isolate. Thus, an actor who is weakly connected (e.g., *k* = 1) will decrease $r_{A}$ more-so than an isolate. In the case of dynamic networks, such as the signal attack in the main text, successful attacks may discourage many weakly connected actors from maintaining or forging ties. Thus, as attacks grow increasingly aggressive, actors who would otherwise have had a low degree centrality become isolates in bulk. Consequently, aggressive attacks can lead to higher assortativity than non-aggressive attacks because they create large numbers of isolates.

The correlation between isolates and assortativity is, however, curvilinear (Fig 5, main text). This is because extremely high levels of intervention splinter the network into numerous distinct components. Moreover, at high levels of intervention, these components will likely not be


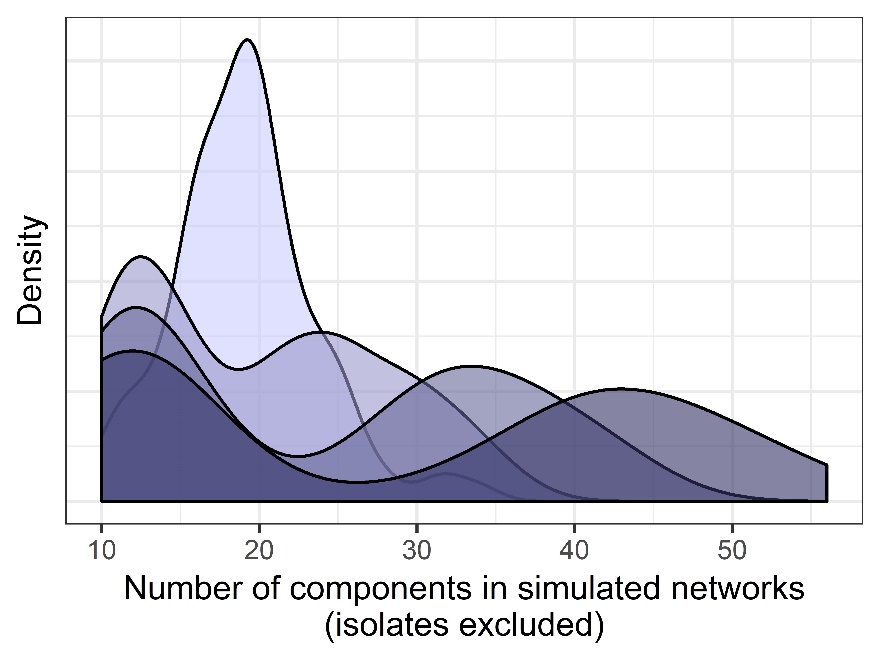


Figure S3. Density plots of non-isolate components in treatment networks (*n* =,1200). Lighter shades correspond to lower levels of intervention, while darker shades correspond to higher levels of intervention.

|  |  |  |  |  |  |
| --- | --- | --- | --- | --- | --- |
|  | | | | |  |
|  | Targeted | Weak link | Signal | Overall |  |
|  | 1 | 2 | 3 | 4 |  |
| Number of isolates | .043^***^ | .039^***^ | .010^*^ | .052^***^ |  |
|  | (.013) | (.007) | (.005) | (.009) |  |
|  |  |  |  |  |  |
| Number of components | -.044^***^ | -.037^***^ | -.008 | -.053^***^ |  |
|  | (.012) | (.008) | (.005) | (.008) |  |
|  |  |  |  |  |  |
| Intercept | -.204^***^ | -.682^***^ | -.626^***^ | -.225^***^ |  |
|  | (.017) | (.102) | (.065) | (.019) |  |
|  |  |  |  |  |  |
| N | 400 | 400 | 400 | 1,300 |  |
| F | 260.1^***^ | 193.0^***^ | 2,944.0^***^ | 130.3^***^ |  |
| R^2^ | .51 | .44 | .92 | .23 |  |
|  | | | | |  |

Table S8. OLS linear models of assortativity by attack strategy. **p*<.05, ****p*<.001. Two-tailed tests. Unstandardized regression coefficients and standard errors for the number of isolates and number of components are multiplied by 10 in all models.

densely interconnected (because of attack damage). Figure S3 demonstrates that higher levels of intervention do indeed tend to have a greater number of non-isolate components (correlation = .43). When attacks generate large numbers of isolates and disconnect components simultaneously, assortativity may decline as the number of isolates increases. Thus, the relationship between the number of isolates and assortativity is only positive after conditioning on the number of components. We illustrate this in Table S8, where we present results from OLS regression. The predictor variables are the number of components and the number of isolates. Results indicate that the number of isolates is positively associated with assortativity in all attack conditions after controlling for the number of components. Inversely, the number of components is negatively correlated with assortativity. Since the number of components and the number of isolates are highly correlated, we perform robustness checks using ridge regression to correct for multicollinearity. The general cross-validation statistic for each ridge regression was approximately zero, indicating that the simple OLS estimates in Table S8 are the best representations of the data. These results illustrate that there is a positive linear association between the number of isolates and assortativity, after conditioning on the number of components.

*References*

1. Duxbury, S.W., Haynie, D. L. (2018). The Network Structure of Opioid Distribution on a Darknet Cryptomarket. *Journal of Quantitative Criminology* 34, 921 – 941*.*
2. Duxbury, S.W., Haynie, D. L. (2018). Building them up, Breaking them down: Topology, vendor selection, and a digital drug market’s robustness to disruption. *Social Networks* 52 (1), 238 – 250.
3. Norbutas, L. (2018). Offline constraints in online drug marketplaces: An exploratory analysis of cryptomarket trade network. *International Journal of Drug Policy* 56, 92 – 100.
4. Snijders, T. A. B. (2017). Stochastic actor-oriented models for network dynamics. *Annual Review of Statistics and Its Application* 4, 343 – 363.
5. Ripley, R. M., Snijders, T. A.B., Boda, Z., Voros, A., Preciado, P. 2017. *Manual for RSiena.* Retrieved May 22^nd^, 2017 (<https://www.stats.ox.ac.uk/~snijders/siena/RSiena_Manual.pdf>).
6. Leifeld, P., Cranmer, S. J., Desmarais, B.J. (2018). Temporal Exponential Random Graph Models with btergm: Estimation and Bootstrap Confidence Intervals. *Journal of Statistical Software* 83(6): 1 – 36.
7. Snijders, T. A. B. (2001). The Statistical Evaluation of Social Network Dynamics. *Sociological Methodology* 31(1): 361 – 395.
8. Snijders, T. A. B., Steglich, C. E. G. (2015). Representing Micro-Macro Linkages by Actor-Based Dynamic Network Models. *Sociological Methods and Research* 44 (1), 222 – 271.
9. adams, j., Schaefer, D. R. (2016). How initial prevalence moderates network-based smoking change: Estimating contextual effects with stochastic actor-based models. *Journal of Health and Social Behavior* 57 (1): 22 – 38.
10. Schaefer, D. R., adams, j., Haas, S.A. (2013). Social Networks and Smoking: Exploring the Effects of Peer Influence and Smoker Popularity through Smoking Simulations. *Health Education and Behavior* 40 (S1), 24 – 32.
11. Bornabeau, E. (2002). Agent-based modeling: Methods and techniques for simulating human systems. *Proceedings of the National Academy of Sciences* 99, 7280 – 7297.
12. Gilbert, N., Troitzsch, K. G. (2005). *Simulation for the Social Scientist* (Open University Press).
13. Groff, E. R., Johnson, S.D., Thornton, A. (2018). State of the Art in Agent-Based Modeling of Urban Crime: An Overview. *Journal of Quantitative Criminology*: 10.1007/s10940-018-9376-y.
14. Barabasi, A. L., Albert, R. (1999). Emergence of Scaling in Random Networks. *Science* 286, 509-512.
15. Clauset, A., Shalizi, C.R., Newman, M.E.J. (2009). Power-law distribution in empirical data. *SIAM Review* 51(4), 661 – 703.
16. Newman, M. E. J. (2002). Assortative mixing in networks. *Physical Review Letters* 89, 208701.
17. Cohen, L. E., Felson, M. (1979). Social Change and Crime Rate Trends: A Routine Activity Approach. *American Sociological Review* 44 (4), 588 – 608.
18. Becker, G. S. (1968). Crime and Punishment: An Economic Approach.” *Journal of Political Economy* 76 (2): 169 – 217.
19. Pogarksy, G., Roche, S. P., Pickett, J. T. (2018). Offender Decision-Making in Criminology: Contributions from Behavioral Economics. *Annual Review of Criminology* 1, 379 – 400.
20. Bushway, Shawn, and Peter Reuter. (2008). Contribution to the Study of Crime and the Criminal Justice System. *Crime and Justice* 37 (1), 389 – 451.
